# Response Surface Methodology Modelling of an Aqueous Two-Phase System for Purification of Protease from *Penicillium candidum* (PCA 1/TT031) under Solid State Fermentation and Its Biochemical Characterization

**DOI:** 10.3390/ijms17111872

**Published:** 2016-11-11

**Authors:** Amaal M. Alhelli, Mohd Yazid Abdul Manap, Abdulkarim Sabo Mohammed, Hamed Mirhosseini, Eilaf Suliman, Zahra Shad, Nameer Khairulla Mohammed, Anis Shobirin Meor Hussin

**Affiliations:** 1 Faculty of Food Science and Technology, University Putra Malaysia, 43400 UPM Serdang, Selangor Darul Ehsan, Malaysia; amaalalhelli@yahoo.com (A.M.A.); myazid@upm.edu.my (M.Y.A.M.); karimsabo@upm.edu.my (A.S.M.); hamedmi@upm.edu.my (H.M.); eilafsuliman@gmail.com (E.S.); mojgan.shad@gmail.com (Z.S.); nameermohammed5@gmail.com (N.K.M.); 2 Institute of technology, Middle technical University, 29008 Alzafaranya, Baghdad, Iraq; 3 Halal Products Research Institute, University Putra Malaysia, 43400 UPM Serdang, Selangor Darul Ehsan, Malaysia

**Keywords:** aqueous two phase systems, *Penicillium candidum*, RSM, protease, solid state fermentation

## Abstract

*Penicillium candidum* (PCA 1/TT031) synthesizes different types of extracellular proteases. The objective of this study is to optimize polyethylene glycol (PEG)/citrate based on an aqueous two-phase system (ATPS) and Response Surface Methodology (RSM) to purify protease from *Penicillium candidum* (PCA 1/TT031). The effects of different PEG molecular weights (1500–10,000 g/mol), PEG concentration (9%–20%), concentrations of NaCl (0%–10%) and the citrate buffer (8%–16%) on protease were also studied. The best protease purification could be achieved under the conditions of 9.0% (*w*/*w*) PEG 8000, 5.2% NaCl, and 15.9% sodium citrate concentration, which resulted in a one-sided protease partitioning for the bottom phase with a partition coefficient of 0.2, a 6.8-fold protease purification factor, and a yield of 93%. The response surface models displayed a significant (*p* ≤ 0.05) response which was fit for the variables that were studied as well as a high coefficient of determination (R^2^). Similarly, the predicted and observed values displayed no significant (*p* > 0.05) differences. In addition, our enzyme characterization study revealed that *Penicillium candidum* (PCA 1/TT031) produced a slight neutral protease with a molecular weight between 100 and 140 kDa. The optimal activity of the purified enzyme occurred at a pH of 6.0 and at a temperature of 50 **°**C. The stability between different pH and temperature ranges along with the effect of chemical metal ions and inhibitors were also studied. Our results reveal that the purified enzyme could be used in the dairy industry such as in accelerated cheese ripening.

## 1. Introduction

Microbial proteases (EC 3.4) are considered as the most important group of industrial enzymes with a great number of industrial and biotechnological applications accounting for about 40% of the total enzyme sales and 60% of the total enzyme market worldwide [1]. The favored source of these biomolecules owes to their rapid growth where the small space is required for cultivation and enabling with which they can be genetically engineered to produce enzymes with customized properties called microorganisms [2]. Synonymies for *Penicillium camemberti* are (*Penicillium album*, *Penicillium caseicolum*, *Penicillium caseicola*, *Penicillium candidum* [3,4]. *P. camemberti* produce two extracellular proteinases [5]: a neutral proteinase and an acid proteinase [6,7,8,9]. *P. camemberti* also synthesis exopeptidases [10]: an exocellular acid carboxypeptidase [11,12], a neutral carboxypeptidase which is mycelial bound [12], and an exocellular aminopeptidase owing to two components [8,13,14,15]. Neutral proteases are significant in the dairy industry since they influence a specific function in hydrolyzing hydrophobic amino acid bonds at a neutral pH, so they reduce the bitterness of food protein hydrolysates [16].

Solid-state fermentation consumes the natural agro-industrial waste as a substrate and thus it is considered a beneficial process [17]. Some studies have aimed at enzyme production by SSF with different organic wastes such as rice and wheat bran, orange peels, soybean meal, banana and apple [18,19]. Nowadays, as an effective green extraction technique, aqueous two phase systems (ATPS) have been used in various processes for purification and recovery of biological products such as enzymes, proteins, nucleic acids, amino acids and microorganisms from contaminants and impurities [20,21,22,23].

ATPS is a very common procedure as it entails a number of benefits over conventional purification techniques used for the production of industrial enzymes because it is easy to scale-up and it offers a non-denaturing environment for biomolecules. ATPS immediately initiates the processing at once by mixing aqueous solutions of a salt and a polymer, or two hydrophilic polymers, beyond a specific critical concentration [24,25]. As both of the phases of ATPS are mostly water-based (80%–85%), ATPS generates a precise condition that makes partitions and concentrates the biomolecules selectively into one of the phases while conserving the essential structure of the biomolecules [20,26]. Polymer–salt systems such as polyethylene glycol (PEG)–magnesium sulfate and PEG–potassium phosphate are among the most commonly used chemicals [27]. However, these inorganic salts lead to high phosphate or sulfate concentration in the waste streams, and consequently affect the environment. So, to degrade the amount of salt discharged into the wastewater is to replace these inorganic salts by citrate, which is recyclable and non-toxic. Though, only a limited amount of investigational effort has been invested in PEG–citrate ATPS [28,29,30].

To optimize the conditions of purification of these enzymes, and to reduce their costs, the amount of work and time, some theoretical tools have been used. Among these, the Response Surface Methodology (RSM) is considered as the gathering of statistical and mathematical analysis that is useful for modeling and analysis in applications where a response of output (or interest) is affected by various factors [31,32,33]. It was previously employed to enhance the recovery and/or the purification of several enzymes [30,34,35].

No current information is available about the ATPS extraction of the extracellular *P. candidum* (PCA 1/TT031) protease. As a result, a study was performed with the objective of handling the ATPS extraction using PEG-citrate–NaCl solution for *P. candidum* (PCA 1/TT031) culture fermentation using RSM and indicating the characteristics of some of its affecting factors.

## 2. Results

### 2.1. Optimizing the Protease Purification Using an Aqueous Two-Phase System

#### 2.1.1. Fitting of the RSM Models

In Table 1, the expected values of the regression coefficients for the RSM models and their corresponding R2 values were described. As it can be seen from the results, the response variables displayed significant (*p* ≤ 0.05) values for the response surface models and a high R^2^ value that was also noted which was in the range of 0.94–0.98. In Table 2, we described the quadratic model and the interactive consequences of the PEG molecular masses (X1), citrate buffer concentration (X3), PEG concentration (X2), and NaCl concentration (X4) for each response variable. Table 2 also describes the significance of the F-ratio and the *p*-value. It can also be seen that the main impact of PEG was retained for all the finally reduced models as they significantly (*p* ≤ 0.05) influenced the responses.

#### 2.1.2. Partition Coefficient (Y1)

**Figure 1:**
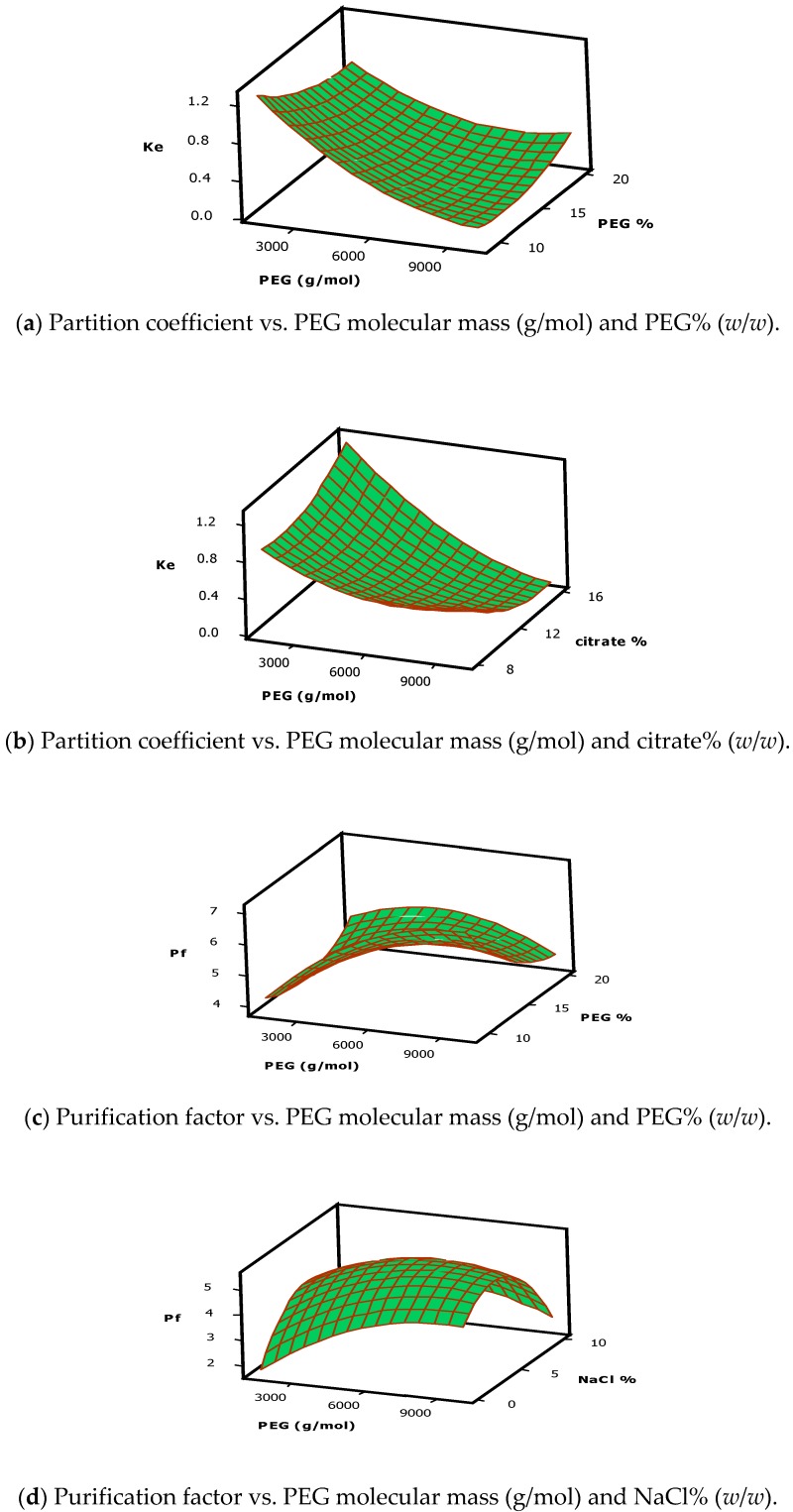

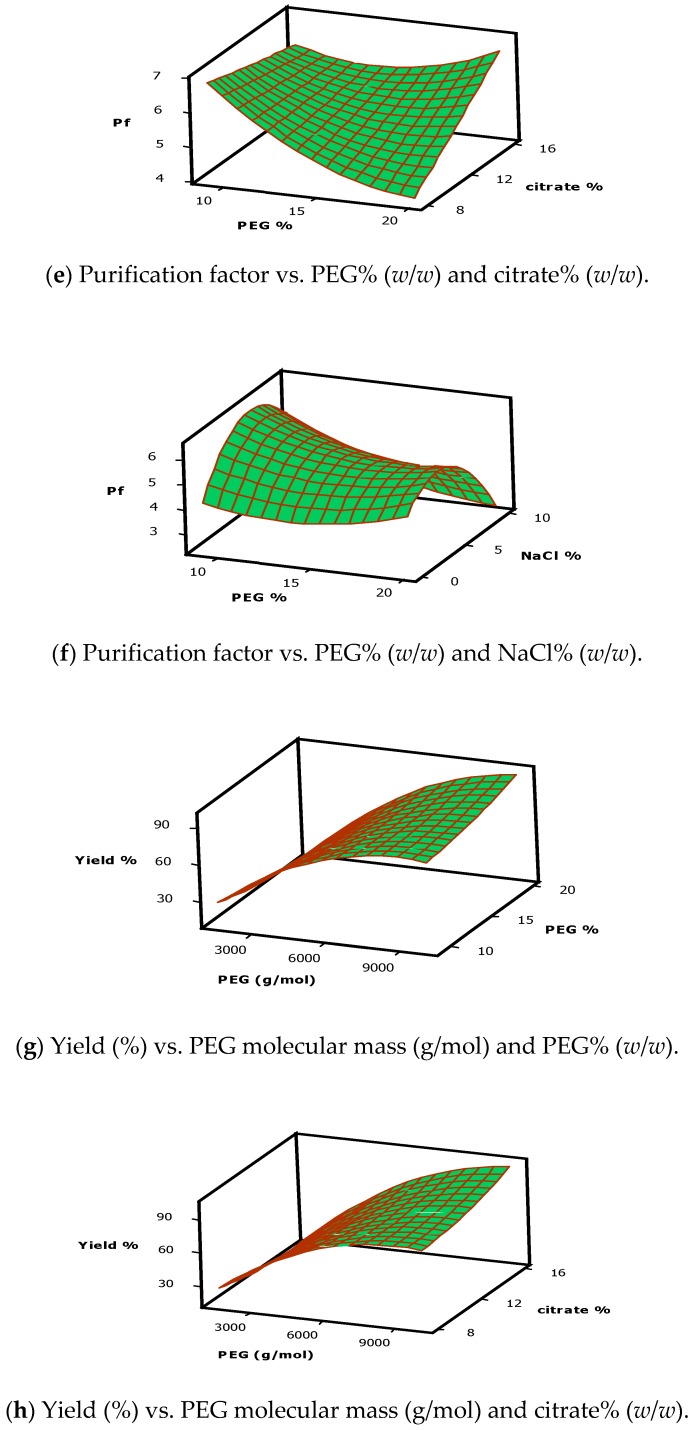

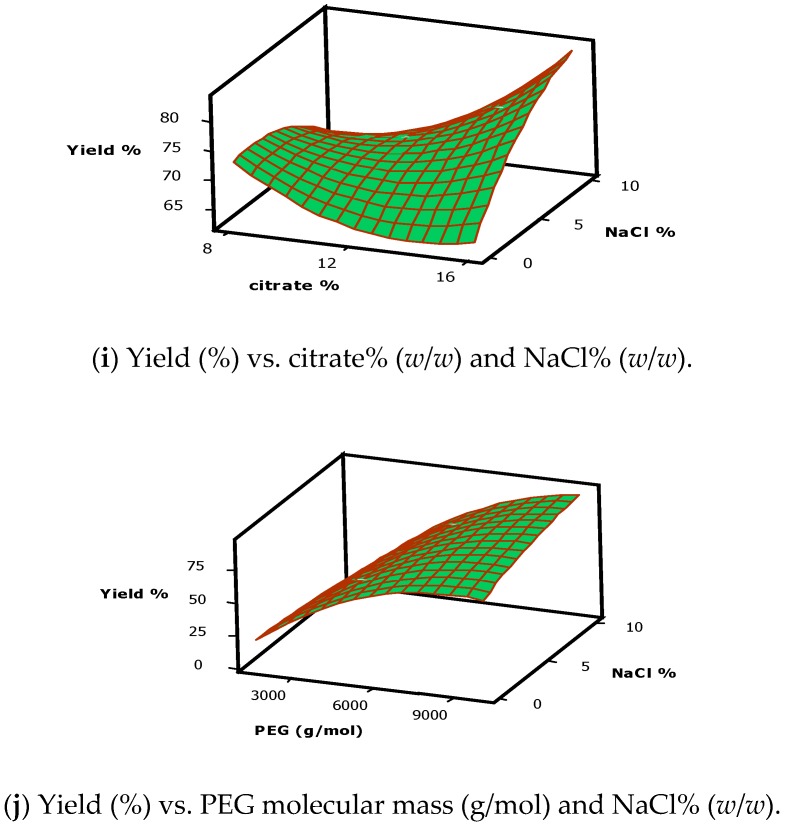
Response surface plots for interaction effects of ATPS purification factors on enzymatic properties of protease. Partition coefficient (**a**–**b**), purification factor (**c**–**f**) and yield (**g**–**j**).

According to the results displayed in Table 2, it can be seen that the response of the partition coefficient (Y1) was heavily and significantly (*p* ≤ 0.05) affected because of the main effects of PEG molecular mass (X1), PEG concentration (X2), sodium citrate buffer concentration (X3), NaCl concentration (X4) and the quadratic effect of the PEG molecular masses (X1^2^), concentration of PEG (X2^2^), concentration of NaCl (X4^2^) and the concentration of citrate buffer (X3^2^), together with the interaction of the PEG molecular mass and its concentration (X1X2), the molecular mass of PEG, and the citrate buffer concentration (X1X3), in the studied ATPS system. Furthermore, it was also observed that the quadratic effect of the NaCl concentration significantly influenced the protease enzyme partition coefficient (Y1) value. It was also seen that the molecular mass of the phase-developing polymer was the main parameter, which greatly affected the ATPS system composition as it could change the phase constituents and alter the polymer and enzyme interaction number [36]. From the results presented in Figure 1a,b, it can be observed that the interactions between the molecular weights of PEG molecules and PEG concentration has led to an increase in the partition coefficient value at the bottom phase as well as an increase in the PEG molecular mass and the concentration of citrate buffer. The optimal protease partition coefficient within the bottom phase (Y1 = 0.2) could be obtained from the following parameters: PEG 8000 (g/mol), a sodium citrate buffer concentration of 15.9% (*w*/*w*), and a PEG concentration of 9.0% (*w*/*w*).

#### 2.1.3. Purification Factor (Y2)

The use of the ATPS system to purify the protease enzyme resulted in purification values (Table 3) that were significantly (*p* ≤ 0.05) impacted by the basic effects of the molecular mass of PEG, the concentration of PEG and NaCl along with the quadratic effects of the molecular weights of PEG, the concentrations of PEG and NaCl and the interactions between the molecular mass and concentration of PEG, PEG molecular mass and the NaCl concentration, concentrations of PEG and citrate buffer, and the concentrations of PEG and NaCl. It was also observed that the volume exclusion effect seen by the PEG 8000 (g/mol) molecules was significantly more profound as compared with the salting out effects [37] and, hence, the protein molecules were seen at the partition mostly in the bottom phase.

Figure 1c–f present the 3-D response surface plots for optimizing the purification factor response at the bottom phase of the ATPS system. Figure 1d presents the contour plots where the elliptical regions in this plot show that the maximal purification factor value for the protease enzyme (Y2 = 6.8) was seen to occur at a molecular mass of PEG 8000 (g/mol) and a concentration of 5.2% (*w*/*w*) NaCl.

The highest purification factor for the protease enzyme (Y2 = 6.8) was calculated as the combined effect of the molecular weight of PEG 8000 (g/mol), PEG concentration of 9.0% (*w*/*w*), concentration of citrate buffer 15.9% (*w*/*w*), and NaCl concentration of 5.2% (*w*/*w*).

#### 2.1.4. Yields (Y3)

In this study, it was noted that the protease enzyme yields were significantly (*p* ≤ 0.05) influenced by the preconditions of the ATPS with respect to the PEG molecular mass and citrate buffer concentration, along with the quadratic effects of the molecular weight of PEG and the interactive effects of the PEG molecular mass and concentration, PEG molecular mass and citrate buffer concentration, PEG molecular weight and NaCl concentration, as well as the citrate buffer and NaCl concentration. The quadratic effects of the PEG molecular mass showed the greatest effect (*p* ≤ 0.05) on protein yields (Y3) (Table 2).

The Figure 1g–j show the interactive effects of the independent variables on the protease yield, whereas the counter curves show the maximal protease yield in the PEG phase. It is also seen that the mid-values for all the factors displayed optimized protease yields. Figure 1h shows the interaction between PEG molecular weight and the concentration of sodium citrate buffer. The maximal protease yield (Y3 = 93%) using the ATPS was achieved under the following conditions—PEG molecular mass of 8000 (g/mol); PEG concentration of 9.0%; citrate concentration of 15.9%; and NaCl concentration of 5.2%.

#### 2.1.5. Experimental Validation of the Models

**Figure 2:**
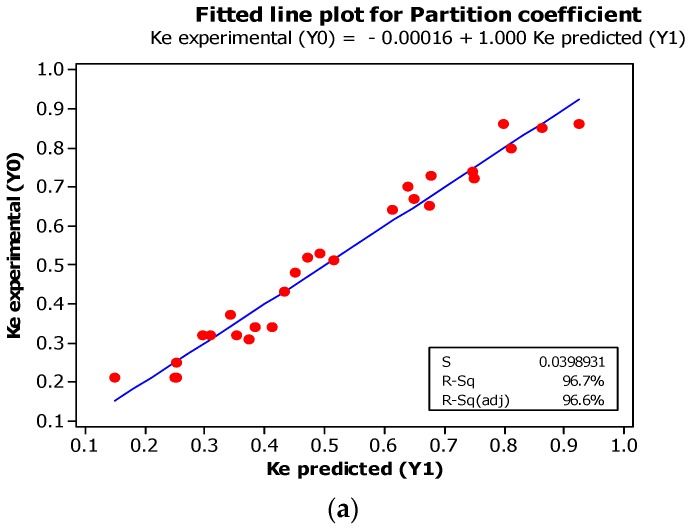

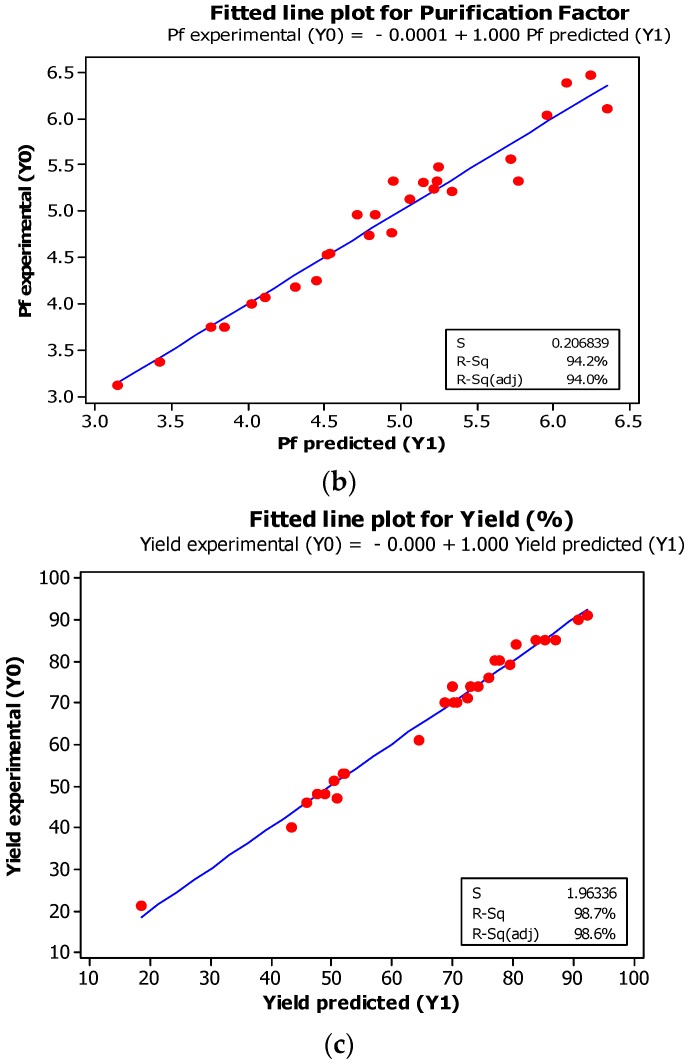
Fitted line plots for predicted (Y1) and experimental values (Y0). Partition coefficient (**a**), purification factor (**b**) and yield % (**c**) of purified protease.

The experimental data and the predicted values were validated for confirmation of the adequacy of the final response surface models. The results should not display any significant difference (*p* > 0.05) and must be in close agreement with the experimental data and the predicted values. In Figure 2, it is shown that the respective values of the response variables obtained from observations were nearer to those estimated in the modelled equations. In this study, the purification scheme was determined and the enzyme biochemical properties were studied. The final model was verified after using the values of PEG molecular weight, concentration of PEG, sodium citrate and NaCl concentration of 8000 (g/mol), 9.0% (*w*/*w*), 15.9% (*w*/*w*), and 5.2% (*w*/*w*), respectively.

### 2.2. Characterization of Protease from P. *candidum* (PCA 1/TT031) in Aqueous Solutions

#### 2.2.1. Influence of Temperature on the Activity and Stability of *P. candidum* (PCA 1/TT031) Protease

**Figure 3:**
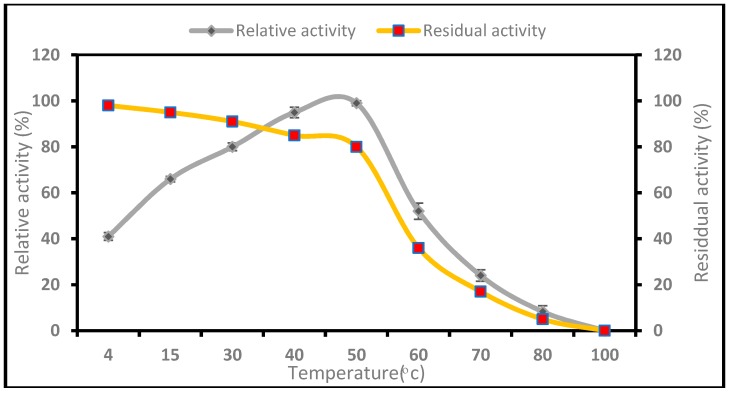
Effect of temperature on protease activity and stability. Error bars represent standard deviation of three replicates.

As shown in Figure 3, the optimum temperature was found to be 50 **°**C and was expressed as a relative activity. The activity of the enzyme was reduced significantly near its half relative activity (52%) by 60 **°**C when the temperature was further increased. This could have been the consequence of the thermal denaturation of protein. The enzyme retained more than 80% of its initial activity at a broad temperature range of 4–50 **°**C. The activity of the *P. candidum* (PCA 1/TT031) was attained and found to be reliant on temperature showing nearly 80%–95% relative activity at 4 **°**C, and 15–50 **°**C.

#### 2.2.2. Influence of pH on the Activity and Stability of *P. candidum* (PCA 1/TT031) Protease

**Figure 4:**
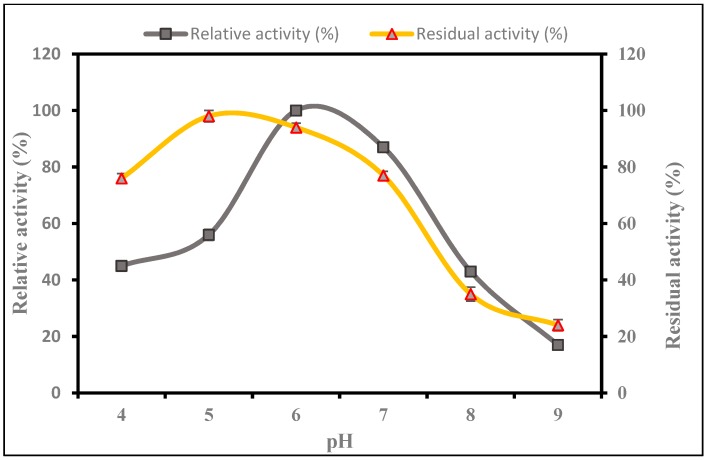
Effect of pH on protease activity and stability. Error bars represent standard deviation of three replicates.

Figure 4 shows that the protease was most active at a pH of 6.0 after which it began to reduce considerably with increasing pH to an alkaline value; it was most stable when held at a pH of 5.0–7.0, preserving more than 77% of its initial activity. However, when incubated at a pH of 8.0 and 9.0 for 1 h, it retained less than 35% of its initial activity probably due to protein denaturation.

#### 2.2.3. Influence of Inhibitors and Metal Ions on the Activity of *P. candidum* (PCA 1/TT031) Protease

The effect of various metallic ions and inhibitors (5, 10 mM) on enzymatic activities is illustrated in Table 3 by adding the relevant captions to the reaction mixture at pH 6.0 and 50 °C. The relative activity levels increased after the purified protease was incubated with 5 mM metallic ions including K^+^, Zn^2+^, Mn^2+^, and Na+, but a slight decrease was seen when the enzymes were incubated with 10 mM metal ion. Additionally, as shown in Table 4, a wholly inhibited response of enzymatic activities was seen when SDS, EDTA, Mg^2+^, Ca^2+^, and Fe^3+^ were present during incubations with both concentrations of inhibitors and metal ions. In contrast, protease was not affected by the serine protease inhibitor PMSF (5, 10 mM).

#### 2.2.4. SDS-PAGE Assessment of Purified Protease and ATPS

**Figure 5:**
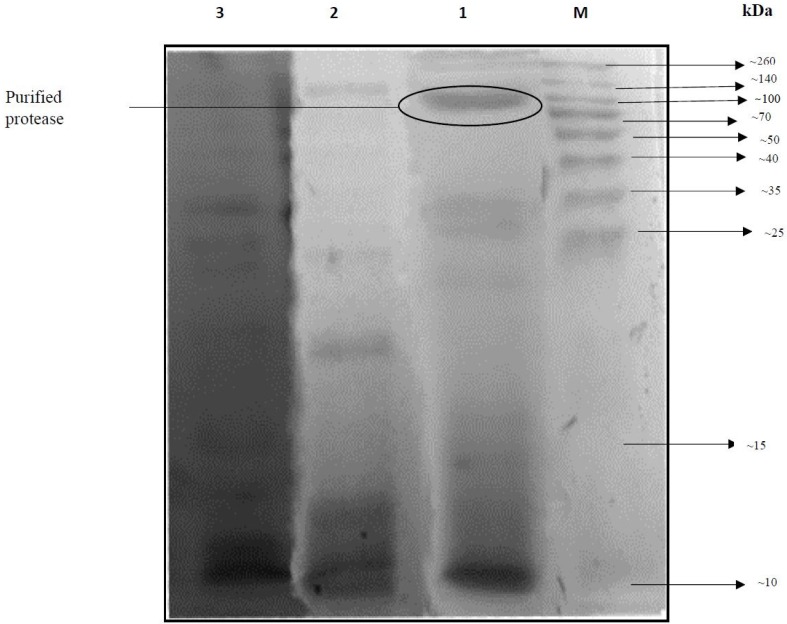
SDS–PAGE profile of *P. candidum* (PCA 1/TT031) protease. Lane M: molecular weight markers (10–260 kDa), lane 1: protease obtained from ATPS bottom phase, lane 2: top phase, lane 3: crude *P. candidum* (PCA 1/TT031) extract.

It was observed that the maximal yields and purification factors of protease from feed stocks were attained in a PEG/citrate/NaCl aqueous two-phase system composed of 8000 (g/mol) molecular masses, 9.0% (*w*/*w*) PEG concentrations, 15.9% (*w*/*w*) citrate buffer concentrations, and 5.2% (*w*/*w*) NaCl concentrations. Purity estimations of the partitioned protease under study were conducted using the SDS-PAGE methods [38]. Figure 5 clarifies the crude feed stocks, which contain ranges of bands representing various molecular masses (Lane 3) that exhibit impurities that are present in the feed stocks. Following ATPS partitioning, the bottom phase sample, which shows protease activity, will present a single dark band (Lane 1).

## 3. Discussion

### 3.1. Protease Purification Using the Aqueous Two-Phase System

#### 3.1.1. Partition Coefficient (Y1)

Figure 1a,b show that the partition coefficient value is lesser than 1; this could be attributed to the fact that an increase in polymer concentration would cause a reduction in the void volume between the polymeric networks causing the molecules present in the above phase to have less space. This is called the “volume exclusion effect” and leads to the biomolecules partitioning at the bottom phase. On the other hand, it was also seen that the biomolecule solubility decreased with an increase in the salt ion concentrations in the bottom (salt-rich) phase, which led to an increase in the partitioning of the biomolecules in the top phase, also known as the “salting out effect” [39,40,41]. Additionally, it is possible that the hydrophilic protease molecules would partition at the salt-rich bottom phase, with an increase in PEG molecular mass, as the hydrophobicity of the PEG molecules increases with an increase in their chain length [42]. When the PEG molecular weight is low, the partition coefficient value is seen to be high and this leads to an increase in the system’s extraction efficiency because of the low excluded volume effect [43].

It was also observed that when low excluded volume effect and the salting out forces are present at the same time, several protein molecules would be partitioned, especially when a low molecular weight PEG concentration is used. The better protein partitioning results which are obtained when using a low PEG molecular weight instead of a higher PEG molecular weight could be due to a significantly lesser interfacial tension at low molecular weight PEG molecules [44]. The partitioning of the protein molecules in the PEG/salt ATPS system can be managed with the help of the PEG volume exclusion effect within the top (or the polymer rich) phase and the salting-out effect within the bottom (salt-rich) phase. It can be seen from the results presented in Figure 1a,b that the interactions between the molecular weights of PEG molecules and the PEG concentration have led to an increase in the partition coefficient value along with an increased PEG molecular mass and concentration of citrate buffer.

The optimal protease partition coefficient within the bottom phase (Y1 = 0.2) could be obtained from the following parameters: PEG 8000 (g/mol), sodium citrate buffer concentration of 15.9% (*w*/*w*), and a PEG concentration of 9.0% (*w*/*w*). This could be due to the volume exclusion effect of the PEG molecules on the biomaterials. Additionally, an increase in the viscosity and compact polymeric network formation could also affect this parameter as a result of using higher molecular weight PEG [37]. Under these circumstances, it has been observed that the proteins favor partitioning in the salt-rich phases or the sediments.

The conformational changes that are seen within the biomolecules and the variations in the molecular weights could greatly affect the exchange between the PEG and the biomolecules, which in response affects the hydrophobic forces which are a result of the presence of other biomolecules in the system. The use of low molecular weight PEG reduces the probability of these effects [45]. Also, the partitioning coefficient has been noticed to enhance with an increase in the concentration of NaCl at the lower phase (salt-rich) because of the generation of higher ionic strength which improves the protein movement to the different phases as a result of the electrostatic repulsion effects. Nascimento et al. [46] observed that the protein which was studied, i.e., a fibrinolytic protease, preferentially partitioned at the bottom phase and displayed a partition coefficient (K) value, which ranged between 0.2 and 0.7, after sodium sulphate and PEG 6000 g/mol were used. Also, Amid, et al. [47] studied the purification process of a purified serine protease enzyme from kesinai leaves using a surfactant–salt ATPS; it was observed that the protein preferentially partitioned at the bottom phase, which was surfactant-rich, whereas the hydrophilic amino acids preferred to partition at the top-phase of the aqueous micellar ATPS. Huddleston, et al. [48] and Barbosa, et al. [49] stated that there is a fine equilibrium present between the hydrophobicity of the PEG molecules and the salting out capability in biomolecular portioning.

#### 3.1.2. Purification Factor (Y2)

One major factor that affects biomolecule partitioning in the ATPS system is the salt concentration. However, we observed that the volume exclusion effect seen with the use of PEG 8000 (g/mol) molecules was significantly more profound as it was compared to the salting out effects [37] and, hence, the protein molecules were seen to have partition at the bottom phase. Thus, the protein purification factor increased because of the exclusion volume effect.

In the ATPS system, the salt concentration is very important for forming an immiscible 2-phase system due to the salting-out effects [50]. However, an increase in salt concentration would also lead to higher viscosity, which would affect the mass transfer [51]. In the study, Nascimento, Sales, Porto, Brandão, de Campos-Takaki, Teixeira, Porto, Porto and Converti [46] investigated the recovery and partial purification of the fibrinolytic protease enzyme from *M. subtilissimus* UCP 1262 with the help of the PEG/sodium sulphate ATPS. It was observed that the protein was preferentially partitioned at the bottom phase. It was further noted that the best enzyme purification was seen when using PEG 6000 g/mol at a concentration of 30.0% (*w*/*w*) and using sodium sulphate at 13.2% (*w*/*w*), leading to a purification factor of 10.0. In contrast, Mehrnoush, et al. [52] found optimum conditions for purification of serine protease from mango peel using the Polyethylene Glycol/Dextran Aqueous Two-Phase System, which was reliant on 8000 g/mol PEG molecular weight, 4.5% NaCl, and 17.2% of tie line length, and a pH of 7.5. This system also provided the highest partition coefficient (84.2), purification factor (14.37), and yield (97.3) under this condition.

Figure 1c–f present the 3-D response surface plots for optimizing the purification factor response at the bottom phase of the ATPS system. Figure 1d presents the contour plots where the elliptical regions in this plot indicate that the maximal purification factor value for the protease enzyme (Y2 = 6.8) occurred at a molecular mass of PEG 8000 (g/mol) and a NaCl concentration of 5.2% (*w*/*w*). It can also be seen that the protease purification factor increased with an increase in the molecular mass of PEG and NaCl concentration, due to the rapid protein partitioning at the bottom phase. Similar to the ATPS purification steps, the highest purification factor for the protease enzyme (Y2 = 6.8) was calculated as a combined effect of the molecular weight of PEG 8000 (g/mol), PEG concentration of 9.0% (*w*/*w*), concentration of citrate buffer of 15.9% (*w*/*w*), and NaCl concentration of 5.2% (*w*/*w*).

#### 3.1.3. Yields (Y3)

An increase in the PEG molecular mass caused an increase in the polymer chain length, thereby resulting in the decrease in the free volume [53]. Hence, the biomolecules prefer to have partition at the bottom phase to improve the protease enzyme yields (Y3). A similar observation was noted which depended on the exclusion-volume effects [54], where a considerable increase in the protease yield was achieved by the addition of PEG, having a molecular mass of 8000 (g/mol). The protease enzyme displayed a very strong propensity to becoming concentrated in the bottom salt-rich phase. This indicates that the protease molecules are very hydrophilic, as the PEG displayed a higher hydrophobicity at the top layer as compared to the bottom layer [55]. Several other researchers reported the same observations for many different enzymes.

Generally, it can be said that positively-charged protein molecules make partition at the bottom phase selectively whereas the negatively-charged protein molecules are mostly seen to distribute in the top phase of the PEG/salt system [55]. In their paper, Karkaş and Önal [37] carried out partitioning of the yeast invertase enzyme via ATPS, under the optimized conditions of (PEG-3000 (15%, *w*/*w*), MnCl_2_ 5% (*w*/*w*), MgSO_4_ (23%, *w*/*w*), at pH 5.0). They achieved a 6.2-fold purification factor and a 217.7% activity recovery for the bottom phase.

Furthermore, Andrews et al. [56] described that the addition of salt would result in the transfer of proteins from one phase to another due to the hydrophobic disparity between the two ATPS phases, protein surface charges, and the hydrophobicity of the proteins. Hence, the NaCl addition was presumed to increase the disparity between the phase hydrophobicity. NaCl could affect protein partitioning because of the differential salt ion distribution between the two separate phases in ATPS. The salt ionic particles displayed differential hydrophobicity, thereby affecting the protein partitioning behavior. The highly hydrophobic ions would then cause the counter ions to make partition towards the more hydrophobic phases while the low hydrophobic ions would like to have partition at the hydrophilic phases [57]. In the presence of salt, the protein yields increased considerably. Hence, salt should be added to the ATPS process to improve protease partitioning.

#### 3.1.4. Experimental Validation of the Models

The final model was verified using PEG molecular weight, concentration of PEG, sodium citrate and NaCl concentration of 8000 (g/mol), 9.0% (*w*/*w*), 15.9% (*w*/*w*), and 5.2% (*w*/*w*), respectively. It was observed that by using this model, the responses of partition coefficient, purification factor, and the protein yield was predicted as 0.2, 6.8, and 93%, respectively, under the optimized conditions. Figure 2 shows that the values for the response variables between the observed and predicted values using the model equations are very close; this proves that the RSM approach is the most suitable methodology for the recovery of desired enzymes.

### 3.2. Characterization of Protease from P. candidum (PCA 1/TT031) in Aqueous Solutions

#### 3.2.1. Influence of Temperature on the Activity and Stability of *P. candidum* (PCA 1/TT031) Protease

The results in Figure 3 are in agreement with other researches. Fuke and Matsuoka [58] purified Prolyl aminopeptidase [EC 3.4.11.5] from the cell-free extract of *P. camemberti* using chromatographic techniques, where the temperature activity of PAP was measured at different temperatures, the maximum temperature of which was 45 **°**C. The enzyme was found to be stable up to 50 **°**C, but the protease activity rapidly decreased at temperatures above 55 **°**C. Moreover, Germano et al. [59] investigated the protease produced by a wild strain of *Penicillium* sp. in a cheaper substrate (defatted soybean cake) and found that the optimum temperature for enzyme activity in the crude extract was 35–45 **°**C. Fuke, Kaminogawa, Matsuoka and Yamauchi [14] purified an extracellular aminopeptidase from *P. caseicolum* using chromatography on DEAE-Sephacel and chromatofocusing on PBE-94 gel. The purified enzyme was most active at 40 **°**C and was stable in a broad range of temperatures of up to 50 **°**C. Additionally, Rodrigues et al. [60] discovered the optimum temperature for the protease from *P. aurantiogriseum* as 50 **°**C. The protease was stable between 25 and 40 **°**C after 2 h incubation. Moreover, nearly 80%–98% relative activity of *P. candidum* (PCA 1/TT031) was achieved at 4 **°**C, 15–50 **°**C, which also indicates that the enzyme might be active at the product ripening temperature (10–15 **°**C).

#### 3.2.2. Influence of pH on the Activity and Stability of *P. candidum* (PCA 1/TT031) Protease

Figure 4 shows that the protease was most active at a pH of 6.0 and was most stable when held at a pH of 5.0–7.0, preserving more than 77% of its initial activity. The optimum pH of protease produced from *P. candidum* (PCA 1/TT031) was 6.0, which indicates that this enzyme is slightly neutral. Agrawal et al. [61] isolated a local strain of *Penicillium* sp. and found that the optimum alkaline protease activity under solid substrate fermentation (SSF) occurred at a pH of 9.0.

Additionally, Germano, Pandey, Osaku, Rocha and Soccol [59] found the optimum pH of a wild strain of *Penicillium* sp. in solid-state fermentation (SSF) to be 6.5 and the enzyme was stable at a pH range of 6.0–9.0. Similarly, Fuke, Kaminogawa, Matsuoka and Yamauchi [14] observed that the *P. caseicolum* aminopeptidase enzyme was most active at a pH of 7.5 and was stable within the pH range of 6.0–7.5. Fuke and Matsuoka [58] noticed that Prolyl aminopeptidase [EC 3.4.11.5] of *P. camemberti* showed the highest activity at pH 7.0.

#### 3.2.3. Influence of Metal Ions and Inhibitors on the Activity of *P. candidum* (PCA 1/TT031) Protease

It was previously stated that the activity of proteases is prompted by many metal ions [62]. As seen in Table 3, the enzyme showed high conformance at a low level of metal ion concentration (5 mM) of K^+^, Zn^2+^, Mn^2+^, and Na^+^ and a slight decrease at a high level of metallic ions. Also, the enzyme was not affected by the addition of PMSF, which indicates that it is not a serine protease. Furthermore, Fuke and Matsuoka [58] found that Prolyl Aminopeptidase from *P. camemberti* would maintain its activity when the enzyme is incubated with Ca^2+^,Co^2+^, Zn^2+^, Mn^2+^ but its activity would be inhibited by Fe^2+^ and Hg^2+^. Fuke, Kaminogawa, Matsuoka and Yamauchi [14] discovered that Mn^2+^ and Zn^2+^ increased the activity of Aminopeptidase I from *P. caseicolum*.

Moreover, Agrawal, et al. [61] detected that the activity of alkaline protease extract from *Penicillium* sp. was drastically inhibited after pre-incubation of the enzyme extract with Fe^3+^, Hg^2+^ and Cu^2+^, whereas Ca^2+^, Mg^2+^ and Mn^2+^ slightly enhanced its activity. Furthermore, Rodrigues et al. [60] noticed that the proteolytic activity of *P. aurantiogriseum* decreased in the amount of Zn^2+^ ion and PMSF and increased in the presence of Mn^2+^. Similarity, Germano et al. [59] reported that the protease produced by *Penicillium* sp. under solid-state fermentation (SSF) was significantly inhibited by incubating the enzyme with an inhibitor agent (5 mM EDTA) and an oxidizing agent.

The presence of Ca^2+^ and K^+^ caused a discrete increase in the proteolytic activity. In review, the protease that agrees with metal ions at higher concentrations would be appropriate in industrial applications such as leather processing, detergent production, sewage treatment, the dairy industry, and other biotechnological applications.

#### 3.2.4. SDS-PAGE Assessment of Purified Protease and ATPS

It was found in previous research that the maximal yields and purification factors of protease from feed stocks could be attained in a PEG/citrate/NaCl aqueous two-phase system which is composed of 8000 (g/mol) molecular masses, 9.0% (*w*/*w*) PEG concentrations, 15.9% (*w*/*w*) citrate buffer concentrations, and 5.2% (*w*/*w*) NaCl concentrations. Purity estimations of the partitioned protease under study were conducted using the SDS-PAGE methods [38]. Figure 5 clarifies the crude feed stocks, which contain ranges of bands representing various molecular masses (Lane 3) that exhibit impurities are present in the feed stocks. Following ATPS partitioning, the bottom phase sample, which shows protease activity, would present with a single dark band (Lane 1), which is evidently the molecular weight of *P. candidum* (PCA 1/TT03) protease, with an estimated value between 100 and 140 kDa. This result is in agreement with the described value from the research of Fuke, Kaminogawa, Matsuoka and Yamauchi [14], which determined the molecular weight of aminopeptidase of *P. caseicolum* to be 120 kDa. Thus, the results of our SDS-PAGE analysis establish that the purification method utilized in this research would result in the maximum recovery of protease from *P. candidum* (PCA 1/TT031).

## 4. Methods and Materials

### 4.1. Materials

All chemicals and reagent were of analytical grade. Wheat bran was purchased from a local shop at Malaysia. Poly ethylene glycol of average molecular weights of 1500, 4000, 6000, 8000 and 10,000 (g/mol) was obtained from Fluka. Co. (St. Louis, MO, USA).

### 4.2. Production of Protease by Solid-State Fermentation

According to a preliminary study of optimization, the condition of gaining high specific activity of crude protease (77.88 U/mg protein) from *P. candidum* ((PCA 1/TT031) commercially freeze-dried strains from Chr. Hansen Sa (Arpajon, France)) was obtained via cultivation the mold in a 10 g wheat bran culture medium with the following ingredients: glucose 3%, yeast extract 5%, moisture ratio 40% and inoculum size 1 × 10^6^ spore/gm wheat bran. The substrate’s initial pH value was regulated to 6 and the medium was incubated at 25 °C.

### 4.3. Enzyme Extraction

Enzyme extraction was achieved after 7 days of fermentation. For this purpose, 50 mL of 1% NaCl was added and stirred using rotary shaker (180 rpm, 30 min) at 37 °C, a temperature high enough to increase the extraction proficiency without causing enzyme denaturation [63]. After that, the samples were filtered through double layer muslin cloth. To isolate the tiny elements of the substrate, both extracts were accumulated and centrifuged (Refrigerated centrifuge SIGMA 3–18K Goettingen, Germany) at 10,000× *g* for 10 min at 4 °C [64]. The clear and brown remaining supernatant was then used directly in the ATPS trials.

### 4.4. Proteolytic Assay

The crude filtrate solution was tested for the protease enzyme activity using the method described by Peres et al. [65]. In this study, azocasein (Sigma Pvt. Ltd., Mumbai, India) was chosen as the substrate due to its high sensitivity, suitability and non-specificity. The substrate was used for the quantification of the proteolytic enzyme activity in the crude extract by determining the release of the red-colored azopeptide. The composition of the assay reaction mixture was as follows: 50 mM Tris-HCI buffer (pH 8.0), 50 µL of azocasein (5 mg/mL, which was prepared in the same buffer), 2 mM CaCl_2_, and crude filtrate extract (50 µL). The total reaction mixture was incubated for 60 min at 37 °C. After 60 min, the reaction was stopped by adding 5% (*w*/*v*) TCA solution (100 µL) and was mixed thoroughly. Then, after 10 min, the assay mixture was centrifuged for 5 min at a speed of 10,000× *g* for 5 min at room temperature. The supernatant (50 µL) was collected from the centrifuged solution and was mixed with 0.5 M NaOH solution (75 µL). This solution contained the low-molecular weight red-colored azopeptides, and they were quantified by determining their absorbance at A450 against the blank.

One protease unit (U) could be defined as the increase of 0.001 in the absorbance value of the solution (in the case of azocasein) per min per mL. The specific activity could be defined as the units (U) per mg of total extracellular proteins.

### 4.5. Protein Determination

Bradford method was used in order to measure the concentration of protein in the samples [66]. As a standard, BSA was used.

### 4.6. Optimizing the Protease Purification Using an Aqueous Two-Phase System

#### 4.6.1. ATPS Preparation

The ATPS was formed by different stock of 50% (*w*/*w*) poly ethylene glycol and 40% (*w*/*w*) sodium citrate solutions. The preparation of phase systems was done in 15 mL graduated centrifuge tubes by weighing an appropriate amount of stock solution of citrate (8%–16%, *w*/*w*) and PEG with various molecular weights (1500–10,000 g/mol), PEG (9%–20%, *w*/*w*) NaCl (0%–10%, *w*/*w*) and 20% (*w*/*w*) crude feedstock. The pH of the citrate solution was regulated to 6.0 by combining required amounts of citric acid monohydrate and tri-sodium citrate dehydrate, following which, an adequate amount of distilled water was added to the system to get a final mass of 10 g system. The ATPS mixture was stirred carefully and centrifuged (Sartorius Model 3-18k, Sartorius AG, Weender Land Strasse, Gottingen, Germany) at 3000 rpm and 25 °C for 10 min, in order to make thermodynamic equilibrium between two phases [67]. Consequently, the two phases became clear and transparent, and the interface became extremely noticeable. By using a pipette, exclusion of the upper phase was carried out and afterwards the lower phase was collected. Protease activity and the volume of each phase was assessed from samples of each phase in conjunction with total protein concentration. The blanks of each system were prepared by the addition of 20% (*w*/*w*) distilled water as a substitute of enzyme in order to minimize interactions of PEG and citrate buffer [68].

#### 4.6.2. Calculations

The partition coefficient (K) of the protease enzyme can be defined to be the ratio of the protease enzyme activity observed in the two phases and could be described with the help of the equation (Equation (1)):

K = A_T_/A_B_ (1)

Additionally, the ratio of the protease specific activity in the bottom phase of the two-phase system, to the primary value of the specific activity in the crude filtrate extract is called as the bottom phase purification fold (P_FT_), and can be represented as (Equation (2)):

P_FT_ = Protease-specific activity of the bottom phase/Specific activity of the crude filtrate solution (2)

Specific activity can be described as the ratio of the total enzyme activity to the overall protein concentration (mg). As the protease enzyme was seen to preferentially partition at the bottom phase, we estimated the total protease yield for the bottom phase as follows (Equation (3)):

Y_B_ (%) = A_B_/A_I_ × 100 (3)

where A_B_ and A_I_ are the protease activities in bottom phase and initial enzyme activity, respectively [32].

#### 4.6.3. Experimental Design

For the determination of the optimized ATPS for purifying the protease enzyme from the filtrate solution, we used the design methodology for finding the experimental space surrounding the preselected conditions. Thereafter, we studied the partition behavior of the protease enzyme and the other contaminating proteins in the solution. We studied the effects of four independent variables, i.e., citrate concentration of buffer (8%–16% (*w*/*w*), X3), PEG concentration (9%–20% (*w*/*w*), X2), PEG molecular mass (1500–10,000 (g/mol), X1), and NaCl concentration (0%–10% (*w*/*w*), X4) and their effect was observed on the purification factor (Y2), the partition coefficient (Y1), and total yield (Y3) for the purified enzyme from the fungus, *P. candidum* (PCA 1/TT031). These variables were optimized using the RSM experimental design. In this study, we have used the Central Composite Design (CCD) for evaluating and estimating the complete quadratic design model for every response. Thirty supernatant samples for all the four variables were assessed with every variable being tested at five different levels (Table 4). The data obtained was evaluated statistically and in a graphical manner by using the statistical software of Minitab v16 (Minitab Inc., State College, PA, USA). The software helped in designing a mathematical design model for determining the likely response for the data obtained.

#### 4.6.4. Variance Analysis

Analysis of Variance (ANOVA) method was used to determine the significant variable of the experiments along with the LSD and examine the least significance tests for measuring the variance between the samples studied. Each sample that was collected was subjected to a 2-fold or a 3-fold estimation and the data was obtained and documented in the form of mean ± SD. An R^2^ value of 0.80 indicated as good model fit [69]. For understanding the actual relationship between the dependent variables (i.e., response factors) and the levels of the independent variables (factors), the polynomial equation that was obtained was then expressed in the form of 3-D surface plots. These 3-D surface plots could be generated by fitting and varying the central point and the two different variables in the experimental ranges. For validating the model and establishing its suitability, the different permutations and combinations of the optimized factors were tested [70].

#### 4.6.5. Optimization and Validation of the Experimental Process

The main aim of optimizing the process is achieving optimal levels for the variables which lead to better response values, and, hence, the individual-level and the popular optimization processes were used for this purpose [71]. Many types of numerical and graphical techniques were used for determining the optimal levels. Additionally, experimental values obtained were compared with the predicted values from the regression equation and the response surface model sufficiency was determined [70,72].

### 4.7. Protease Characterization

#### 4.7.1. Influence of Temperature on Activity and Stability of *P. candidum* (PCA 1/TT031) Protease

To study the effect of temperature on the enzyme activity from the salt-rich bottom phase, the protease activity was determined at the temperatures of 4, 15 and 30–100 °C (interval 10 °C). Similarly, the thermostability of the enzyme was analyzed at a temperature of 4 and 30–100 °C (intervals of 10 °C), and the residual activity was measured after an incubation period of 1 h. Protease assay was then conducted for the incubated samples.

#### 4.7.2. Influence of pH on Activity and Stability of *P. candidum* (PCA 1/TT031) Protease

The effect of pH value on the enzyme activity was researched by determining the enzyme activity from the salt-rich bottom phase at 50 °C using different buffers within the pH value range of 4.0–9.0 (50 mM sodium citrate buffer with pH 4.0 and 5.0; 50 mM potassium phosphate with pH 6.0 and 7.0; and 50 mM Tris-HCl with pH 8.0 and 9.0). Additionally, enzyme pH stability was investigated by incubating the enzyme at 50 °C for 1 h using buffers within the pH value range of 4.0–9.0 (50 mM sodium citrate buffer with pH 4.0 and 5.0; 50 mM potassium phosphate with pH 6.0 and 7.0; and 50 mM Tris-HCl with pH 8.0 and 9.0).

#### 4.7.3. Influence of Metal Ions and Inhibitors on Activity of *P. candidum* (PCA 1/TT031) Protease

Chloride salts of metal ions such as, K^+^, Zn^2+^, Mn^2+^, Na^+^, Ca^2+^, Co^+2^, Fe^2+^, SDS, EDTA and PMSF were dissolved at a concentration of 5 mM and 10 mM in deionised water. The effect of metal ions and inhibitors on the enzyme activity from the salt-rich bottom phase was studied by incubating equal volumes of these solutions and purified enzyme in a water bath at 30 °C for 1 h and then the residual activity was measured using the optimum assay.

#### 4.7.4. SDS–PAGE Analysis

To assess the purity of protease, SDS–PAGE was applied, according to Laemmli [38]. The acrylamide gel used in this study established of 12%, 4.5% of resolving gel and stacking gel, respectively. A 0.05% (*v*/*v*) Coomassie^®^ Brilliant Blue G-250 (Sigma) was utilized to stain the gel.

## 5. Conclusions

In this report, to investigate the main effects as well as the interaction effects of the variables that are important in the extraction of protease from *P. candidum* (PCA 1/TT031), RSM was conducted. The regression equations which were obtained were significant (*p* ≤ 0.05), and had high R^2^ > 0.80. The results suggested that the optimum purification conditions of the protease from *P. candidum* (PCA 1/TT031) were gained using 8000 (g/mol) PEG molecular weight, 9.0% (*w*/*w*) PEG concentration, 15.9% (*w*/*w*) citrate concentration and 5.2% (*w*/*w*) NaCl. Under the optimum conditions, the partition coefficient, purification factor and yield of the protease were 0.2, 6.8 and 93%, respectively. The enzyme, which was characterized as neutral, demonstrated an optimal temperature for protease activity of 50 °C, at which it was totally stable, and increased its activity in the presence of some metal ions (K^+^, Zn^2+^, Mn^2+^, and Na^+^ ) or deceased its activity in the presence of others (Mg^2+^, Ca^2+^, Fe^3+^ SDS and EDTA). This is the first study on protease purification using an aqueous two-phase system and the results are likely to be encouraging for the dairy industry.

## Figures and Tables

**Table 1:** Regression coefficients, R^2^, adjusted R^2^ and probability value of the response surface models.

Regression Coefficient	Partition Coefficient (Y1)	Purification Factor (Y2)	Yield (Y3)
b0	4.25371	8.00997	122.231
b1	−0.00019	0.000124	0.007
b2	−0.18962	−0.72459	−4.359
b3	−0.20842	−0.61406	−10.700
b4	−0.14749	0.99890	−2.721
b1^2^	0.00000	−0.00000	−0.000
b2^2^	0.00426	0.01864	0.099
b3^2^	0.01023	0.00242	0.281
b4^2^	0.01415	−0.07165	−0.140
b12	0.00001	−0.00004	0.000
b13	−0.00001	0.00001	0.000
b14	0.00000	−0.00003	0.000
b23	0.00170	0.03580	0.023
b24	−0.00082	−0.02791	−0.127
b34	0.00063	0.02063	0.350
R^2^	0.96	0.94	0.98
R^2^ (adj.)	0.92	0.87	0.97
Regression (*p-*value)	0.000 ^a^	0.000 ^a^	0.000 ^a^

b0, b1, b2, b3 and b4: The estimated regression coefficient for the main linear effects. b1^2^, b2^2^, b3^2^ and b4^2^: The estimated regression coefficient for quadratic effects. b12, b13, b14, b23, b24 and b34: The estimated regression coefficient for the interaction effects. 1: Molecular mass of PEG; 2: PEG concentration; 3: citrate concentration; 4: NaCl concentration; ^a^ significant (*p* ≤ 0.05).

**Table 2:** The significance of each independent variable effect indicated by using F–ratio and *p-*value in the final models.

Ariables	Main Effects					Quadratic Effects				Interaction Effects					
	Independent Variable	*X1*	*X2*	*X3*	*X4*	*X1^2^*	*X2^2^*	*X3^2^*	*X4^2^*	*X1X2*	*X1X3*	*X1X4*	*X2X3*	*X2X4*	*X3X4*
Partition coefficient (Y1)	*p*-value	0.010 ^a^	0.005 ^a^	0.024 ^a^	0.015 ^a^	0.000 ^a^	0.013 ^a^	0.003 ^a^	0.000 ^a^	0.016 ^a^	0.002 ^a^	0.432	0.533	0.707	0.834
	F-ratio	9.05	11.24	6.55	7.82	26.16	8.29	13.40	62.56	7.70	14.44	0.66	0.41	0.15	0.05
Purification factor (Y2)	*p*-value	0.002 ^a^	0.028 ^a^	0.170	0.003 ^a^	0.005 ^a^	0.030 ^a^	0.870	0.000 ^a^	0.016 ^a^	0.670	0.040 ^a^	0.022 ^a^	0.025 ^a^	0.197
	F-ratio	14.00	6.11	2.12	13.35	11.65	5.91	0.03	59.96	7.61	0.19	5.20	6.73	6.39	1.85
Yield %(Y3)	*p*-value	0.037 ^a^	0.141	0.019 ^a^	0.313	0.000 ^a^	0.196	0.062	0.136	0.016 ^a^	0.022 ^a^	0.011 ^a^	0.865	0.246	0.030 ^a^
	F-ratio	5.39	2.45	7.13	1.10	66.90	1.86	4.18	2.53	7.71	6.78	8.70	0.03	1.48	5.90

*X1*, *X2*, *X3* and *X4*: The main effect of PEG molecular mass, PEG concentration, citrate concentration and NaCl, respectively; *X1^2^*, *X2^2^*, *X3^2^* and *X4^2^*: The quadratic effect of PEG molecular mass, PEG concentration, citrate concentration and NaCl, respectively; *X1X2*: The interaction effect of PEG molecular mass and PEG concentration; *X1X3*: The interaction effect of PEG molecular mass and citrate concentration; *X1X4*: The interaction effect of PEG molecular mass and NaCl; *X2X3*: The interaction effect of PEG concentration and citrate; *X2X4*: The interaction effect of PEG concentration and NaCl; *X3X4*: The interaction effect of buffer concentration and NaCl; ^a^ significant (*p* ≤ 0.05).

**Table 3:** Effect of metal ions on protease activity from *P. candidum (*PCA 1/TT031).

Reagent	Relative Activity (% ± SD) a	
	Concentration 5 mM	Concentration 10 mM
Without component	100 ± 0.00 d	100 ± 0.00 b
NaCl	136 ± 1.7 a	112 ± 2.5 a
ZnCl_2_	122 ± 2.5 b	92 ± 2.0 c
KCl	112 ± 2.0 c	85 ± 3.0 d
MnCl_2_	110 ± 2.0 c	98 ± 2.0 b
MgCl_2_	44 ± 2.6 f	31 ± 1.5 e
CaCl_2_	34 ± 2.0 g	23 ± 1.5 f
FeCl_3_	25 ± 3.00 h	0.00 ± 0.00 g
Sodium dodecyl sulfate (SDS)	54 ± 3.5 e	33 ± 2.5 e
Ethylenediaminetetraacetate (EDTA)	15 ± 2.0 i	7 ± 2.0 h
PMSF (Phenylmethanesulfonylflouride)	100 ± 1.3 d	100 ± 1.7 b

Means with the same letter are not significantly difference (*p* > 0.05).

**Table 4:** The matrix of the central composite design (CCD).

Run Order		Independent Variable			
	Block	PEG Molecular Mass (g/mol,X1)	PEG Concentration (*w*/*w*, X2)	Citrate Concentration (*w*/*w*, X3)	NaCl Concentration (*w*/*w*, X4)
1	3	6000	20	12	5
2c	3	6000	14.5	12	5
3	3	1500	14.5	12	5
4	3	6000	14.5	16	5
5	3	10,000	14.5	12	5
6	3	6000	9	12	5
7	3	6000	14.5	12	10
8	3	6000	14.5	12	0
9	3	6000	14.5	8	5
10 ^c^	3	6000	14.5	12	5
11 ^c^	1	6000	14.5	12	5
12 ^c^	1	6000	14.5	12	5
13	1	4000	17.25	14	7.5
14	1	8000	17.25	10	7.5
15	1	8000	11.75	10	2.5
16	1	8000	11.75	14	7.5
17	1	8000	17.25	14	2.5
18	1	4000	11.75	10	7.5
19	1	4000	11.75	14	2.5
20	1	4000	17.25	10	2.5
21 ^c^	2	6000	14.5	12	5
22	2	4000	17.25	10	7.5
23	2	8000	17.25	10	2.5
24	2	8000	11.75	14	2.5
25	2	8000	17.25	14	7.5
26	2	4000	11.75	14	7.5
27	2	4000	11.75	10	2.5
28	2	8000	11.75	10	7.5
29	2	4000	17.25	14	2.5
30 ^c^	2	6000	14.5	12	5

^c^ Center point.
